# Comprehensive in situ mapping of human cortical transcriptomic cell types

**DOI:** 10.1038/s42003-021-02517-z

**Published:** 2021-08-24

**Authors:** Christoffer Mattsson Langseth, Daniel Gyllborg, Jeremy A. Miller, Jennie L. Close, Brian Long, Ed S. Lein, Markus M. Hilscher, Mats Nilsson

**Affiliations:** 1 Science for Life Laboratory, Department of Biochemistry and Biophysics, Stockholm University, Solna, Sweden; 2 Allen Institute for Brain Science, Seattle, WA USA

**Keywords:** Cell biology, Cellular neuroscience

## Abstract

The ability to spatially resolve the cellular architecture of human cortical cell types over informative areas is essential to understanding brain function. We combined in situ sequencing gene expression data and single-nucleus RNA-sequencing cell type definitions to spatially map cells in sections of the human cortex via probabilistic cell typing. We mapped and classified a total of 59,816 cells into all 75 previously defined subtypes to create a first spatial atlas of human cortical cells in their native position, their abundances and genetic signatures. We also examined the precise within- and across-layer distributions of all the cell types and provide a resource for the cell atlas community. The abundances and locations presented here could serve as a reference for further studies, that include human brain tissues and disease applications at the cell type level.

## Abstract (web summary)

Langseth et al. present cell type maps of human cortical tissue sections and show an efficient and robust workflow to accurately resolve anatomical organization of human brain tissue.

## Introduction

The human cortex contains roughly 80 billion cells^1,2^ and understanding the identity of all the cells is challenging. Evolving single-cell technologies have recently allowed scientists to start to comprehend the cellular composition of the human cortex^3–5^, enabling quantitative descriptions of cellular diversity and definitions^6^. Hodge and colleagues characterized 15,928 cell nuclei from the human middle temporal gyrus (MTG, Brodmann area 21, a part of the temporal lobe) by single-nucleus RNA-sequencing (snRNA-seq)^4^. However, the precise laminar locations and abundances of many of these cell types have not yet been described, and beyond coarse layers most of these types are at low proportions and intermingled with one another. Hybridization-based in situ sequencing (HybISS) is an image-based multi-targeted gene expression profiling technique that allows the precise mapping of individual cells in human brain tissues^7^. Various analytical approaches can assign detected transcripts to segmented cells and subsequently, cells to cell types. One such approach, probabilistic cell typing by in situ sequencing (pciSeq), leverages single-cell RNA-sequencing data to guide cell type assignment^8,9^. Here, we implement pciSeq to map cell types across three human cortical sections as a proof of principle to show an efficient and robust method to accurately resolve anatomical organization of human tissue that is envisioned for such efforts as the Human Cell Atlas^10^.

## Results

**Fig. 1:**
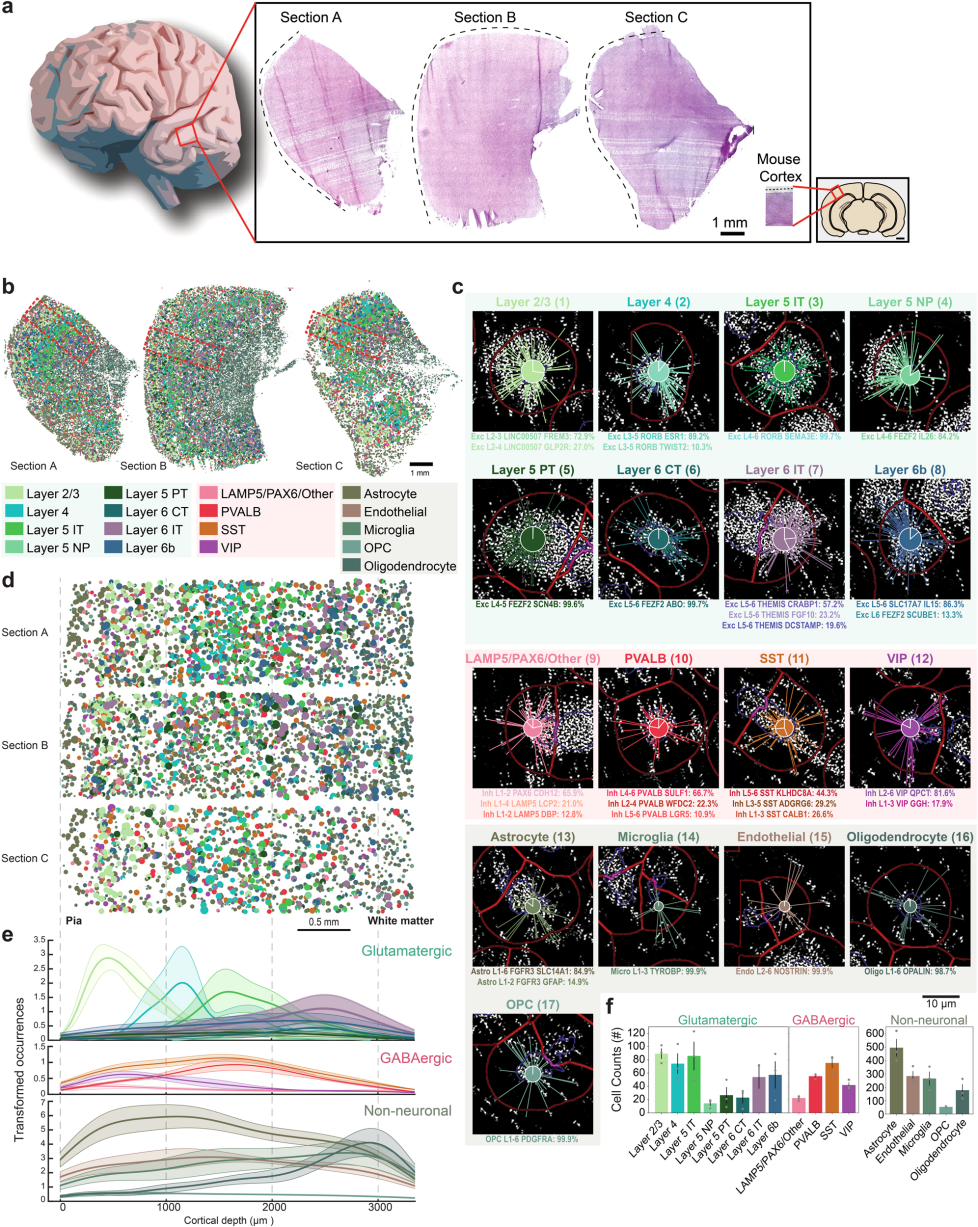
Cell types of the human temporal lobe. **a** Three tissue sections from the temporal lobe (Sections A–C) and the mouse visual cortex for size reference. **b** Spatial maps of cell types across human temporal lobe, including 24 glutamatergic, 45 GABAergic and 6 non-neuronal cells. The cells are represented as pie charts where the angle of each slice is proportional to the likelihood of the cell being of a certain type and the size of the pie chart is indicative of the number of transcripts. The colors correspond to the cell subclasses stated below the maps. Red rectangles mark the regions of interest (ROIs) in **d**. **c** Genes are assigned to cells, and cells are subsequently classified based on single-nucleus RNA-sequencing (snRNA-seq) data. Examples shown are median cells for each cell subclass, i.e. cells with the median count of the number of transcripts being assigned within each subclass. The number next to the cell subclass label denotes the cell location in Supplementary Fig. 2. The probability for each cell type is listed below the example cells. **d** ROIs spanning across the neocortical layers in the tissue sections in **a**. **e** Mean cortical depth profiles from the ROIs, with the transparent shades representing the standard error of the mean. *Y*-axis represents occurrences after smoothening data with a kernel (100 bins). Separated histograms for each cell subclass are shown in Supplementary Fig. 6. **f** Mean counts of each cell subclass found within the ROIs (error bars represent standard error of the mean, *n* = 3 tissue sections).

As part of a Human Cell Atlas pilot project to explore cell-type mapping with spatial transcriptomics methods (the SpaceTx consortium), we obtained human temporal lobe tissue from surgical resections (Fig. 1a). Using a panel of 120 genes chosen to span snRNA-seq cell type definitions in the MTG, we applied pciSeq to produce cellular maps of human brain tissue. We mapped 59,816 cells of 75 transcriptomic cell types including 24 glutamatergic, 45 GABAergic, and 6 non-neuronal cell types (Section A: 19,127 cells, 27.51 mm^2^; Section B: 28,694 cells, 41.41 mm^2^; Section C: 11,995 cells, 30.23 mm^2^; Fig. 1a, b; Supplementary Fig. 1). pciSeq assigns cells, represented as pie charts, with the angle of each slice proportional to the cell type probability. For instance, a cell of subclass Layer 2/3 can have a 72.8% probability of being an Exc L2–3 *LINC00507 FREM3* cell type and 27.2% being an Exc L2–3 *LINC00507 GLP2R* cell type (Fig. 1c; Supplementary Fig. 2). Here, the highest probability in the pie chart defines the final cell type for all downstream analysis. The level of certainty for each cell subclass is shown in Supplementary Fig. 3. The size of each pie chart is indicative of the number of assigned transcripts. On average, 70.9 ± 7.4 transcripts and 30.4 ± 2.0 distinctive genes were measured in neuronal cells, approximately twice as many as non-neuronal cells (Supplementary Fig. 4). Counting the cell type occurrences in our tissue sections shows that non-neuronal cell types outnumber neuronal cell types by 3.38, comparable to published results that measured a ratio of 3.76 over the entire human cortex^1^.

To accommodate for tissue variability and size as tissue sections were from surgical interventions, regions of interest (ROIs) were outlined spanning the six neocortical layers and some white matter (Fig. 1d; Supplementary Fig. 5a). First, we investigated the distribution of the assigned cell types as a function of cortical depth, measured from the pial surface (Fig. 1e; Supplementary Fig. 6). Glutamatergic cells often showed characteristic depth profiles corresponding to cortical layers while GABAergic cells were less confined. However, the VIP subclass and the LAMP5/PAX6/Other subclass had the highest density in supragranular layers, whereas the PVALB subclass and SST subclass peaked around the granular layer, similar to what has been shown in mouse neocortex^11,12^. Among the non-neuronal cells, oligodendrocytes were most distinct, showing a higher cellular density in infragranular layers and white matter. Counting the occurrence of neuronal cell types showed a proportion of 67:33 for glutamatergic versus GABAergic cells. Layer 2/3, Layer 4, Layer 5 IT, and Layer 6b cells were the most abundant cell types in the glutamatergic population and SST and PVALB cells in the GABAergic population (Fig. 1f).

**Fig. 2:**
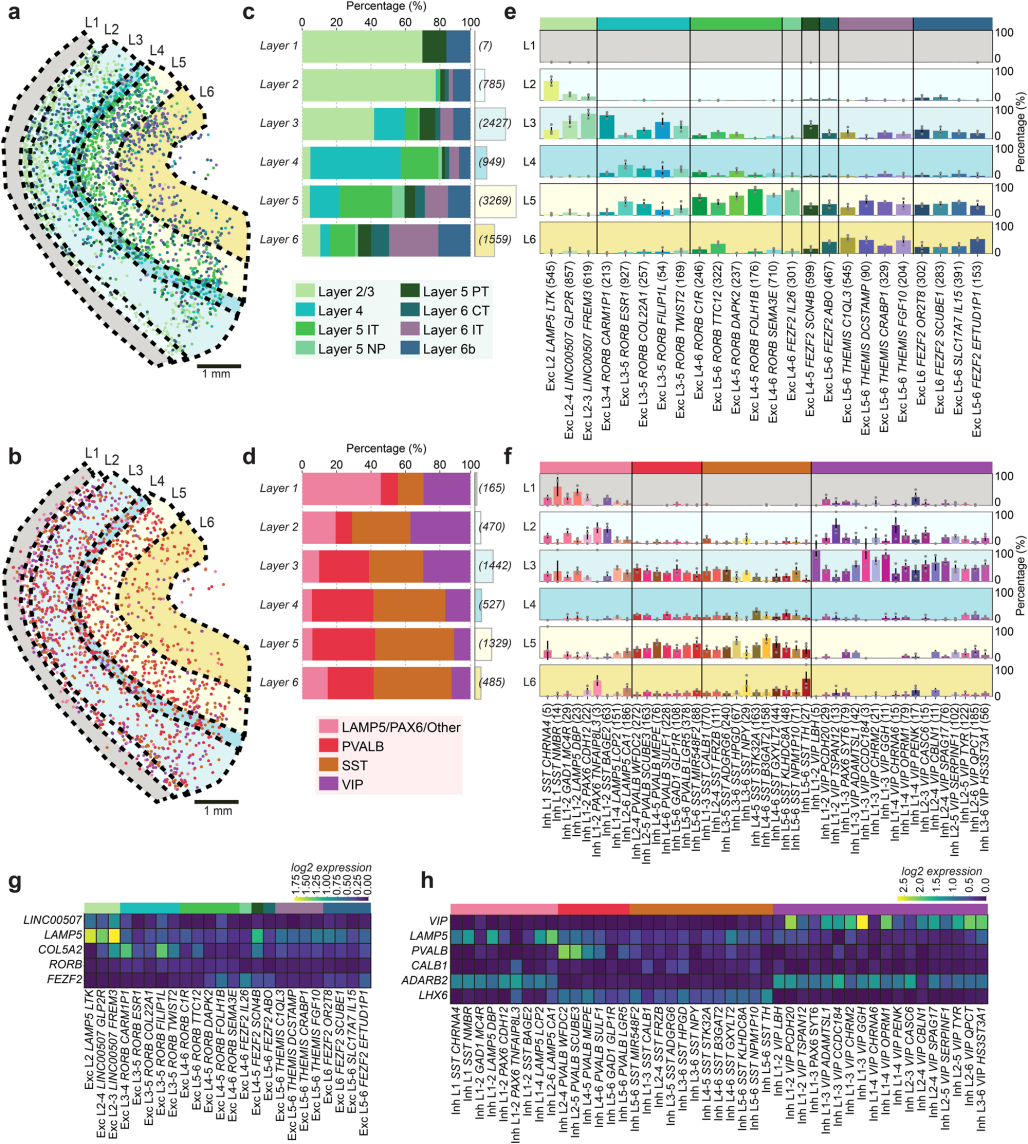
Layer-specificity and molecular composition of neurons in the human temporal lobe. **a** Location of glutamatergic cells colored by the most probable cell subclass and annotated neocortical layers (L1–6). **b** Same as in **a** for GABAergic cells. **c** The within-layer relative distribution of the glutamatergic cells and the number of cells counted for each layer in brackets. **d** Same as **b** for the GABAergic cells. **e** Across-layer distribution of glutamatergic cell types. The colored bars represent the relative proportion of each cell type in each layer (L1–6). Error bars represent the standard error of the mean (*n* = 3 tissue sections). **f** Same as **e** for GABAergic cell types. **g** Mean log2-transformed expression of known glutamatergic marker genes. **h** Same as **g** for GABAergic marker genes.

To further examine the neocortical architecture, we manually demarcated the six cortical layers guided by known layer markers (*LAMP5*, *LINC00507, COL5A2, FEZF2*) and estimated published layer thickness^13^ (Fig. 2a, b; Supplementary Figs. 5b, 7 and 8a). We quantified the ratio of cell types within and across layers. Within-layer distribution of glutamatergic cells largely followed the proposed layer locations of Hodge et al.^4^ (Fig. 2c). For example, 79% of mapped cells in layer 2 were classified as Layer 2/3 cells whereas 53.7% of mapped cells in layer 4 were classified as Layer 4 cells. Compared to glutamatergic neurons, GABAergic neurons (Fig. 2d) and non-neuronal cells (Supplementary Fig. 8b) were more homogeneously distributed. These trends followed the snRNA-seq data (Supplementary Fig. 9a–c). Across layers, we further separated the eight glutamatergic subclasses into the 24 cell types, the four GABAergic subclasses into 45 types, and the five non-neuronal subclasses into 6 types (Fig. 2e, f; Supplementary Fig. 8c). Glutamatergic cell types of supragranular and granular layers (L1–3 and L4, respectively) showed clear layer structure (Fig. 2e), and similar across-layer distributions as the snRNA-seq data (quantified with Pearson correlation coefficient; Supplementary Figs. 9f and 10a). The mean correlation coefficient was 0.77 ± 0.05 for glutamatergic cells, which is in a similar range as the sample-to-sample correlation of glutamatergic cells in our pciSeq data (mean Pearson correlation coefficient: 0.85 ± 0.03). Considering the layer-specificity of the genes that define these cell types, we noted that cells in supragranular and granular layers were well defined by the genes *LINC00507* and *COL5A2* respectively and infragranular layers (L5–6) were mainly defined by *FEZF2* (Fig. 2g; Supplementary Fig. 11a). GABAergic cell types were sparser than glutamatergic cell types, with LAMP5/PAX6/Other and VIP cells escalating in supragranular layers and SST and PVALB cells around the granular layer (Fig. 2f). Comparing the differences in layer distribution of cells mapped with pciSeq and snRNA-seq, the mean Pearson correlation coefficient was 0.63 ± 0.04 (Supplementary Figs. 9e and 10b). The mean sample-to-sample correlation of GABAergic cells in our pciSeq was 0.67 ± 0.01, exposing more variability and less profound laminar distributions compared to glutamatergic cells. However, many cell types followed the proposed cell type location by Hodge et al.^4^, such as the LAMP5 cell types. Also, *ADARB2* (a marker for CGE-derived cells, such as LAMP5 and VIP cells) and *LHX6* (a marker for MGE-derived cells, such as PVALB and SST cells) showed a clear separation between cells in supragranular layers versus cells in infragranular layers (Fig. 2h). Similar to glutamatergic cells, marker gene expression was not entirely restricted to layers. While *VIP* gene expression was most abundant in supragranular layers, it could also be found in infragranular layers (Supplementary Fig. 11b). Among the cell types in the non-neuronal population, there was a notable increase in the density of cells in infragranular layers and white matter (Supplementary Figs. 7c and 8a). The oligodendrocytes (Oligo L1-6 *OPALIN*) exhibited a distinct layer distribution being more abundant in the infragranular layers (Supplementary Fig. 8c). This was consistent with the principal marker for the oligodendrocytes, *OPALIN* as it was mostly expressed in the infragranular layers and white matter (Supplementary Figs. 8d and 11c). Quantifying the difference in layer distribution between pciSeq and snRNA-seq showed lower agreement (0.35 ± 0.18 mean Pearson correlation coefficient) to the snRNA-seq (Supplementary Figs. 9f and 10c) and could reflect the difference in a sampling of snRNA-seq data, that contains 94% neuronal cells vs. 6% non-neuronal cells. Azevedo et al.^1^ suggest 20% neuronal vs. 80% non-neuronal cell types in the cerebral cortex. pciSeq results show comparable quantities for neuronal (23%) vs. non-neuronal (77%) cells and also a sample-to-sample correlation of non-neuronal cells in pciSeq data was high (0.83 ± 0.06). Examining abundances for neuronal cells showed that decreased abundances in pciSeq data were on average 1.5% (25 GABAergic types) and 4.6% (8 glutamatergic cell types) less abundant compared to snRNA-seq. Cells with increased abundances in pciSeq data were on average 1.9% (20 GABAergic types) and 2.3% (16 glutamatergic cell types) more abundant, confirming the currently known proportions for neuronal cell types.

**Fig. 3:**
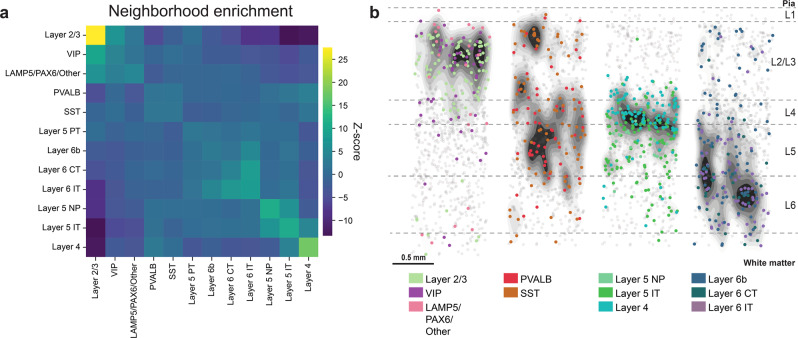
Neighborhood enrichment analysis of human cell types. **a** Neighborhood enrichment analysis for neuronal subclasses of all three tissue sections (shown as mean, *n* = 3 tissue sections). Cluster proximity of subclasses is quantified with a permutation-based test (1000 permutations), by comparing the actual cell type labels with a random configuration of labels and maintaining the positional information. From each pair (actual label vs. label of each permutation), the means and standard deviations are estimated, and a *Z*-score is calculated. The *Z*-score indicates if a cluster pair is over-represented or over-depleted in the analysis. **b** Maps on top of density plots (black) for subclasses from neighborhood enrichment analysis in **a**, highlighting the increased spatial proximity of the subclasses.

Next, we analyzed the spatial proximity of the neuronal subtypes in the tissues (Fig. 3a) and performed a neighborhood enrichment analysis^14^. This analysis quantifies cluster proximity with a permutation-based test, i.e. it compares the cell type labels with a random configuration of labels by maintaining the positional information and counting the number of cells recovered in each iteration. Our analysis showed four main enrichments. Among the glutamatergic subclasses, Layer 5 NP cells showed enrichment with Layer 5 IT and Layer 4 cells. Layer 6b cells were enriched with Layer 6 CT and Layer 6 IT cells. Among the GABAergic subclasses, VIP cells showed enrichment with LAMP5/PAX6/Other cells and SST cells with PVALB cells, given their preferential occurrences in supragranular and granular layers, respectively (Fig. 3b). Similarly, Layer 2/3 cells exposed neighborhood enrichment with supragranular interneurons, while Layer 5 PT cells grouped with granular interneurons.

Since pciSeq also allows cell-type mapping of snRNA-seq data sets that have not been used for gene panel selection, we further applied this method using an independent snRNA-seq data set from Lake et al.^15^. Three excitatory clusters (Ex2, Ex3, Ex7) in the snRNA-seq data by Lake et al.^15^ matched one-to-one with clusters in Hodge et al.^4^ and the remaining clusters mapped to multiple clusters (see Supplementary Fig. 12c). Plotting the locations for the individual cells of Lake et al.^15^ and coloring them by the most probable cell subclass shows clear differences of within- and across-layer distributions of cell types (Supplementary Fig. 12). However, the laminar distributions were not as fine as with the snRNA-seq data by Hodge et al.^4^, that includes 24 glutamatergic cell types (Fig. 2e). Still, while the absolute abundance of the 14 matched cell types was reduced compared to the absolute abundance of the corresponding cell types in Fig. 2 (3255 cells vs. 5147 cells or 14 cell types vs. 24 cell types for Section A), the relative abundances over the cortical layers show large agreements between these two cell type maps. This highlights that pciSeq comprehensively maps human cortical cell types and even allows for fine-tuning when targeting more cell types with deeper snRNA-seq data.

## Discussion

Taken together, we mapped 24 glutamatergic, 45 GABAergic, and 6 non-neuronal cell types previously classified by snRNA-seq^4^. Within the targeted gene list, classical markers for glutamatergic, GABAergic and non-neuronal cell types were included to aid in probabilistic assignment of cells in situ. However, some classical markers such as *SST*, a main marker in the human cortex, were not prioritized by the gene selection approach. Therefore, *SST* subclass genes were used for cell calls. The combinatorial detection approach of the genes allows and assures the precise assignment of molecularly defined cell classes as well as their subtypes in situ. Even fine cell types can be typed, such as the Sst + Chodl + cell type (Inh L3-6 SST NPY), a well-characterized but rare cell type^16,17^.

Our spatially resolved, transcriptomically profiled tissue sections display a similar non-neuronal to neuronal ratio as the previously reported, immunocytochemically measured ratio in the entire cerebral cortex^1^. Comparison to published sequencing data also suggests a high correlation in the layer distribution between in situ data and snRNA-seq^4^ data. Here, manual layer annotations were guided by four known marker genes but layer outlines could also be identified by cellular granularity or more automated approaches^18,19^ which take into consideration that layer boundaries are not sharp. We found mainly glutamatergic cell types to arrange in layers but they were less layer-restricted than expected^4^, advocating that anatomical location alone is not sufficient to characterize a cell.

pciSeq presents itself as a powerful tool in cell type assignment across large tissue areas, not only for In situ sequencing data but also other spot-based spatial approaches^20,21^. The presented human cortical maps include a comprehensive reference of the cells in the human temporal lobe, their spatial location, abundances, and gene expression signatures, all central features outlined by the Human Cell Atlas^10^ and others^6^. This work embodies the vision and paves the path towards a spatial Human Cell Atlas^10^, utilizing the predefined taxonomy of cells^4^ to create maps of histological tissue structures.

## Methods

### Tissue description

Tissue procurement from neurosurgical donors was performed outside of the supervision of the Allen Institute at local hospitals, and tissue was provided to the Allen Institute under the authority of the IRB of each participating hospital. A hospital-appointed case coordinator obtained informed consent from donors prior to surgery. Tissue specimens were de-identified prior to receipt by Allen Institute personnel. The specimens collected for this study were apparently non-pathological tissues removed during the normal course of surgery to access underlying pathological tissues. Tissue specimens collected were determined to be non-essential for diagnostic purposes by medical staff and would have otherwise been discarded. Section A and C were from the MTG of a 38-year old male epilepsy donor. Section B was from the frontotemporal parietal lobe of a 72-year-old male tumor case.

### Tissue preparation

Tissue collection and processing were performed as previously described in Hodge et al.^4^, in accordance with the provisions of the United States Uniform Anatomical Gift Act of 2006 described in the California Health and Safety Code section 7150 (effective 1 January 2008) and other applicable state and federal laws and regulations. In short, surgically resected temporal lobe tissue was divided into 500 μm slices using an EMS TC-1 tissue chopper. The 500 μm sections were then further sliced to 10 μm and collected on slides. Slices were placed on the flat bottom surface of a cryo-embedding mold and embedded in OCT. After 15 min of equilibration in OCT, slices were frozen in a dry ice—ethanol slurry, vacuum sealed, and stored at −80 °C. Tissue sections for HybISS experiments were received from the Allen Institute under the SpaceTx consortium and all ethical regulations were followed.

### HybISS

Technical details and descriptions of the HybISS method can be found published in Gyllborg et al.^7^ as well as transcript identity and coordinates for Sections A and B. The same gene panel and experimental procedures were used in the generation of Section C. In brief, after fixation sections were permeabilized with 0.1 M HCl for 5 min and washed with PBS. mRNA was reverse transcribed priming with random decamers, RNase inhibitor, and reverse transcriptase (BLIRT) overnight at 37 °C. Tissue sections were then fixed for 40 min post reverse transcription and subsequently washed with PBS. Phosphorylated padlock probes (PLPs) were hybridized at a final concentration of 10 nM/PLP and ligated in the same reaction with Tth Ligase (BLIRT) and RNaseH. This was performed at 37 °C for 30 min and then moved to 45 °C for 1.5 h. Sections were washed with PBS and RCA was performed with phi29 polymerase (BLIRT) and Exonuclease I (Thermo Fisher Scientific) overnight at 30 °C. The sections were treated with TrueBlack Lipofuscin Autofluorescence Quencher (TLAQ) (Biotium) for 45 s and immediately washed with PBS. Bridge-probes (10 nM) were hybridized at RT for 1 h in hybridization buffer (2× SSC, 20% formamide), followed by hybridization of readout detection probes (100 nM) and DAPI (Biotium) in the hybridization buffer for 2 h at RT. The genes targeted in the HybISS experiments (capturing arms of PLPs) were manually and computationally curated as a part of the Chan Zuckerberg Initiative SpaceTx Consortium. The panels and the computational selection were based on snRNA-seq data from human MTG^4,22^. The main computational algorithms used were NSforest^23^, ProMMT^24^, and mfishtools^25^. Imaging was performed using a standard epifluorescence microscope (Zeiss Axio Imager.Z2) connected to an external LED source (Lumencor^®^ SPECTRA X light engine) as described in Gyllborg et al.^7^.

### Image analysis

The same in-house software as in Gyllborg et al.^7^ was used for the generation of the spatial gene expression data and is available at https://github.com/Moldia.

### Cellular segmentation

Cells were segmented using CellProfiler 2.2.0^26^ in which the diameter of the objects was set to fit the span of nuclei sizes. The DAPI images were tiled to reduce the computational requirements. A manual threshold was set for localizing the nuclei. The localized objects were then expanded and subsequently converted to images. The area and location of the objects were recorded.

### pciSeq

The pciSeq pipeline can be found at https://github.com/acycliq/full_coronal_section and is described in Qian et al.^8^. The pciSeq pipeline works by assigning genes to cells and then cells to cell types. This assignment is done using a probabilistic framework based on single-cell RNA sequencing data. The automated workflow consists of top-hat filtering of HybISS and DAPI images, initial nonlinear image registration, spot detection, fine image registration, crosstalk compensation, gene calling, and cell calling. Thresholding for spot detection was initially optimized in Qian et al.^8^ and depends on the signal-to-noise ratio, i.e. the spot intensity and the averaged intensity around the spot. Here, the threshold was adjusted manually, as HybISS signal-to-noise ratio and spot intensity after top-hat-filtering are on average two times higher than the signal-to-noise ratio in Qian et al.^8^. DAPI images were segmented with standard watershed segmentation. The input data for cell calling consists of the area, the global *x* and *y* coordinates, and the unique cellular identifier of the segmented cells. In addition, global *x* and *y* coordinates of the transcript, the transcript type, and the cell that the transcript belongs to are needed for each transcript. Cell type definitions used in pciSeq were downloaded from the Allen Brain Atlas (https://portal.brain-map.org/atlases-and-data/rnaseq). All of the colors used in the figures follow the cell type color definition formulated by the Allen Institute.

### pciSeq processing module

The downstream processing of the data (available upon request) was done using a set of functions created in-house (https://github.com/Moldia/pciseq_processing_module). The set of tools included finding the most probable cell type call from the pciSeq output, plotting these cell type calls, plotting the cell types individually, adding polygon labels to the cells, and creating cell-by-gene matrices of the called cells.

### ROI and layer annotations

The ROIs were drawn using napari^27^, a multi-dimensional image viewer for python. The ROIs were outlined to cover the six neocortical layers and some white matter. The area of each ROI was the same for all tissue sections (3.2 mm^2^). The layers were annotated in apart based on the expression of known marker genes (*LAMP5*, *LINC00507*, *COL5A2*, *FEZF2*) in addition to the relative thickness of cortical layers^13^.

### Statistics and reproducibility

For the pciSeq to snRNA-seq correspondence analysis, the mean relative layer distribution was calculated for each cell type in the pciSeq data and the snRNA-seq data, where the layer distribution was extracted from the metadata from Hodge et al.^4^. The Pearson *r* correlation coefficient was then calculated on the laminar distributions per cell type and plotted on the *x*-axis. The relative occurrences of cell types were plotted on the *y*-axis (for glutamatergic, GABAergic, and non-neuronal cells separately), subtracting relative snRNA-seq occurrences from relative pciSeq occurrences for each cell type. The mean correlation coefficient was calculated for pciSeq vs. snRNA-seq and also the sample-to-sample correlation was calculated for the three tissue sections.

### Neighborhood enrichment analysis

The neighborhood enrichment analysis was performed using squidpy^14^. The connectivity matrix was computed for each section by itself and then compiled by calculating mean scores.

### Reporting summary

Further information on research design is available in the Nature Research Reporting Summary linked to this article.

## Supplementary information

Supplementary Information

Description of Supplementary Files

Supplementary Data 1

Reporting Summary

## Data Availability

Single-nucleus RNA-sequencing cell type definitions are from Hodge et al.^4^ and can be accessed through the Allen Brain Atlas data portal at https://portal.brain-map.org/atlases-and-data/rnaseq. Cell metadata for the cell maps and figures are provided as Supplementary Data. Cell-by-gene matrices are available upon request.

## Code availability

The code and software used to generate the plots for the Figures is available at the following repository: https://github.com/Moldia/spatial_mapping_of_human_cortical_cells
