# Supplementary material for: Comprehensive in situ mapping of human cortical transcriptomic cell types
Source: Commun Biol. 2021 Aug 24;4:998. doi: 10.1038/s42003-021-02517-z (PMC8384853; doi:10.1038/s42003-021-02517-z)
Supplement: Supplementary file 1 — Supplementary Information [file 42003_2021_2517_MOESM1_ESM.pdf]

Supplementary Figures

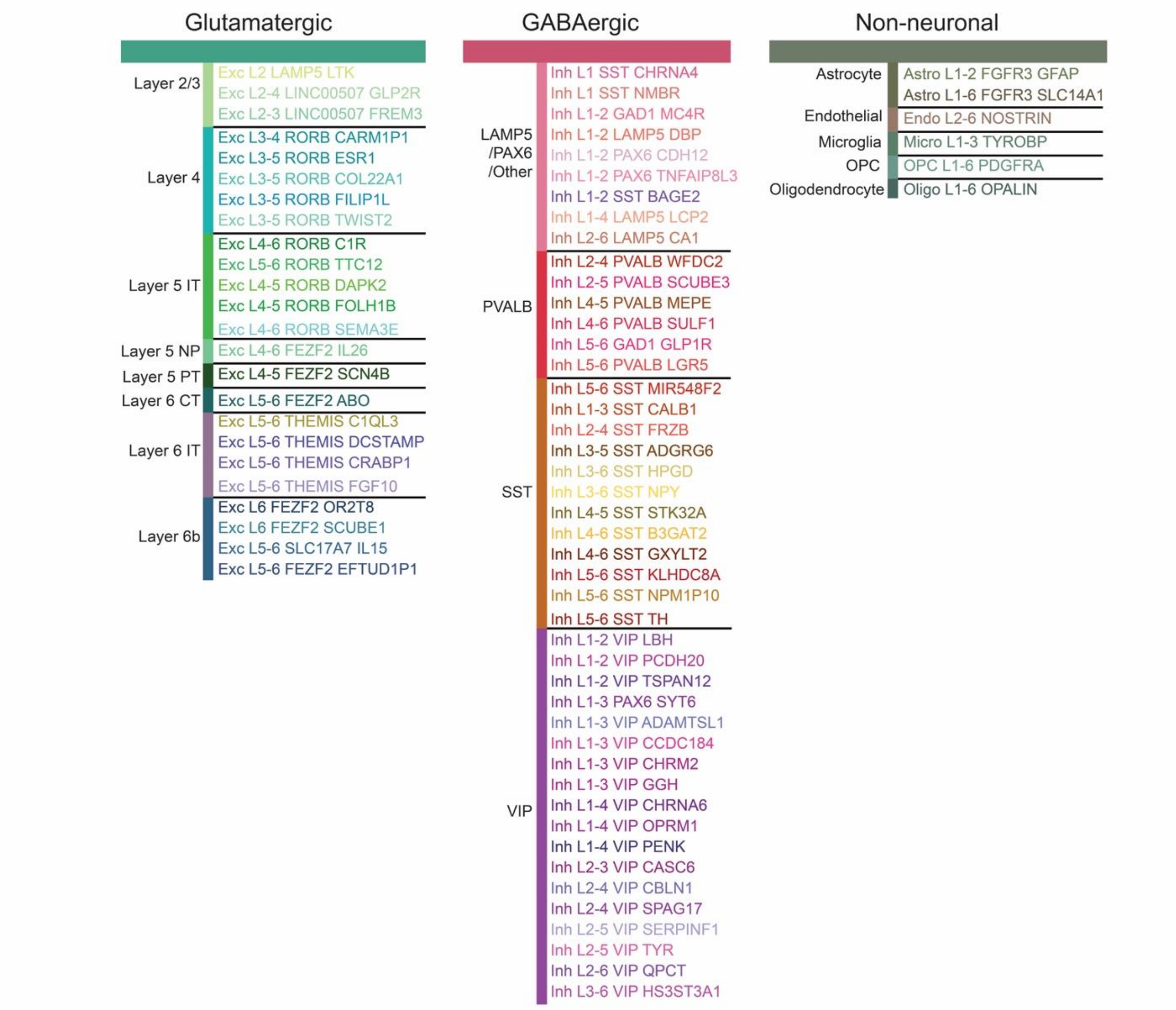

Supplementary Figure 1. Cellular hierarchy and cell type definitions.

The top level is the separation into glutamatergic, GABAergic and non-neuronal cells. The second level is the separation of the glutamatergic, GABAergic and non-neuronal cells into 17 subclasses. The third level is the separation of the 17 subclasses into 75 cell types.

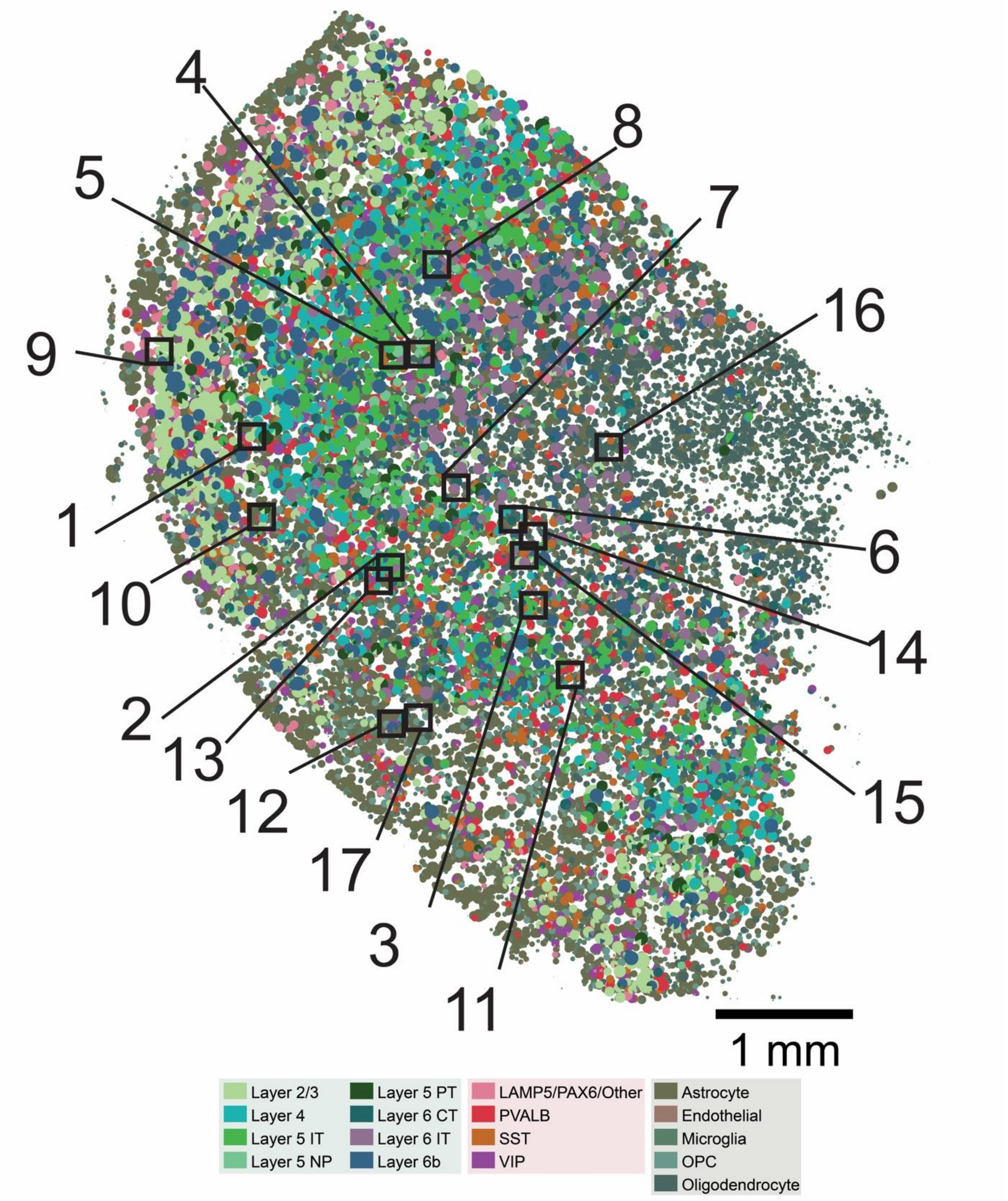

Supplementary Figure 2. Approximate location of example cortical cells.

The location of the example cells shown in **Figure 1c.** The colors correspond to the 17 cell subclasses. Black boxes represent the approximate location of each cell and the number denotes the corresponding cell.

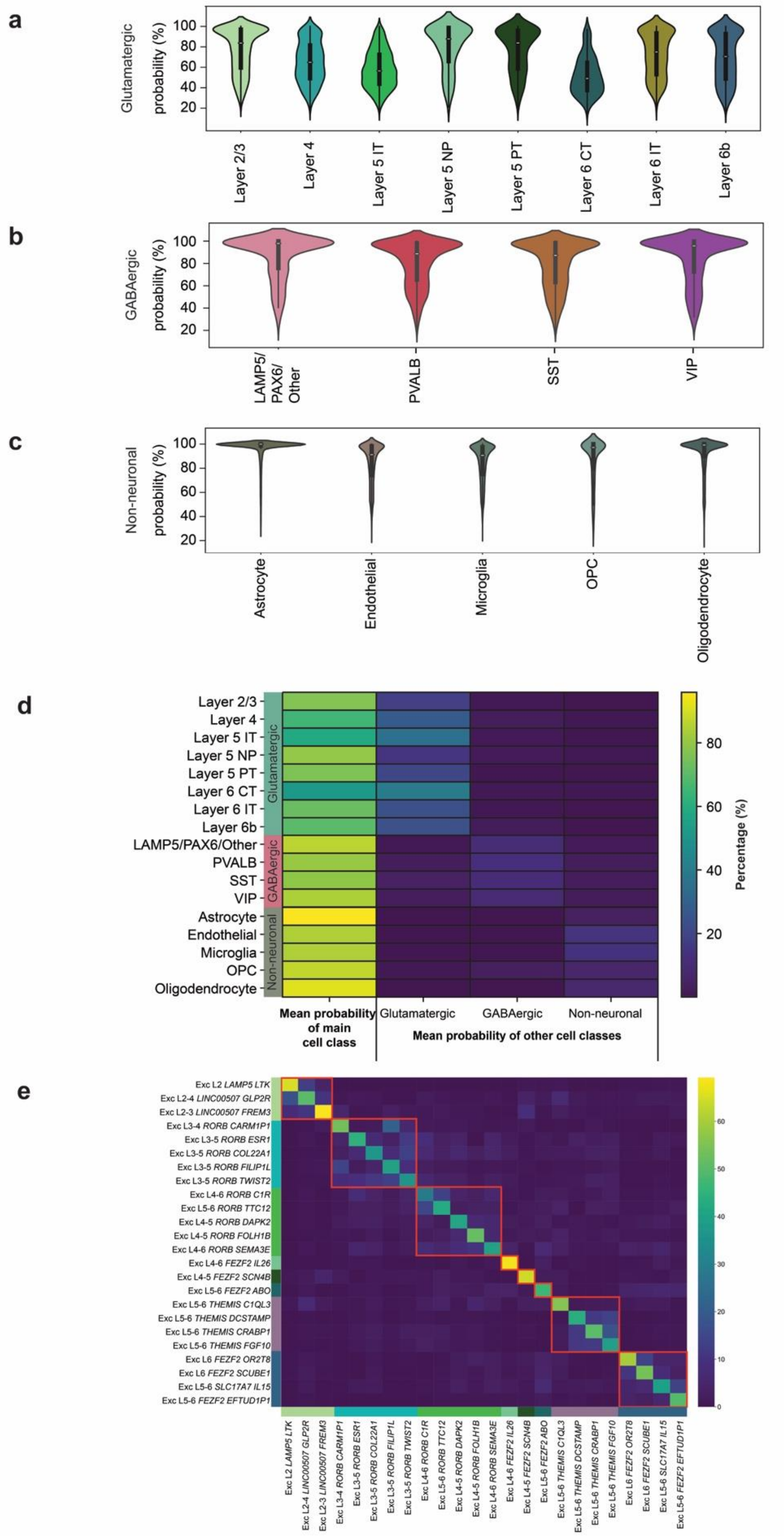
a
Glutamatergic probability (%)
100
80
60
40
20
Layer 2/3
Layer 4
Layer 5 IT
Layer 5 NP
Layer 5 PT
Layer 6 CT
Layer 6 IT
Layer 6b
b
GABAergic probability (%)
LAMP5/PAX6/Other
PVALB
SST
VIP
c
Non-neuronal probability (%)
Astrocyte
Endothelial
Microglia
OPC
Oligodendrocyte
d
Glutamatergic
GABAergic
Non-neuronal
LAMP5/PAX6/Other
Percentage (%)
Mean probability of main cell class
Mean probability of other cell classes
e
Exc L2 LAMP5 LTK
Exc L2-4 LINC00507 GLP2R
Exc L2-3 LINC00507 FREM3
Exc L3-4 RORB CARM1P1
Exc L3-5 RORB ESR1
Exc L3-5 RORB COL22A1
Exc L3-5 RORB FILIP1L
Exc L3-5 RORB TWIST2
Exc L4-6 RORB C1R
Exc L5-6 RORB TTC12
Exc L4-5 RORB DAPK2
Exc L4-5 RORB FOLH1B
Exc L4-6 RORB SEMA3E
Exc L4-6 FEZF2 IL26
Exc L4-5 FEZF2 SCN4B
Exc L5-6 FEZF2 ABO
Exc L5-6 THEMIS C1QL3
Exc L5-6 THEMIS DCSTAMP
Exc L5-6 THEMIS CRABP1
Exc L5-6 THEMIS FGF10
Exc L6 FEZF2 OR2T8
Exc L6 FEZF2 SCUBE1
Exc L5-6 SLC17A7 IL15
Exc L5-6 FEZF2 EFTUD1P1
60
50
40
30
20
10
0

Supplementary Figure 3. Level of certainty for each cell subclass.

a, Violin plots showing data density, with median and 1.5 times the interquartile range of the highest probability in each pie chart from pciSeq for glutamatergic cells. The mean certainty for glutamatergic cells is 68.42 ± 0.23%. **b,** Same as **a** for GABAergic cells with a mean certainty of 81.42 ± 0.31%. **c,** Same as **a** for non-neuronal cells with a mean certainty of 89.38 ± 0.07%. **d,** Heatmap of the probability distribution in the pie charts for each cell subclass. Data are represented as mean. The mean of the highest probability (largest pie) for each cell subclass is highlighted in the first column. The mean of the other probabilities (smaller pies) are shown in the remaining three columns and grouped for glutamatergic cells, GABAergic cells and non-neuronal cells. This highlights that cells mostly share pie charts with cell types of the same subclass, i.e. glutamatergic cells with other glutamatergic cells, GABAergic cells with other GABAergic cells and non-neuronal cells with other non-neuronal cells. **e,** Detailed heatmap of the probability distribution in the pie charts for each glutamatergic cell type. Red boxes highlight the cell types of the same subclass.

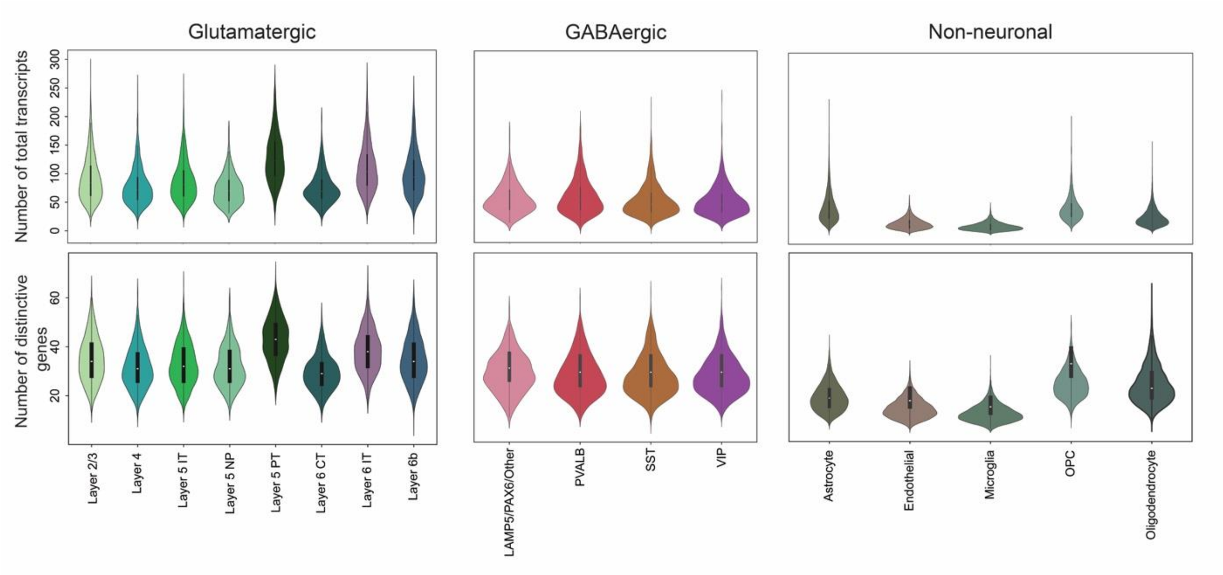

Supplementary Figure 4. Transcript and gene quantification in single cells.

Top: Violin plots showing data density, with median and 1.5 times the interquartile range of the number of total transcripts in glutamatergic, GABAergic and non-neuronal cells. Bottom: The number of distinct genes in glutamatergic, GABAergic and non-neuronal cells.

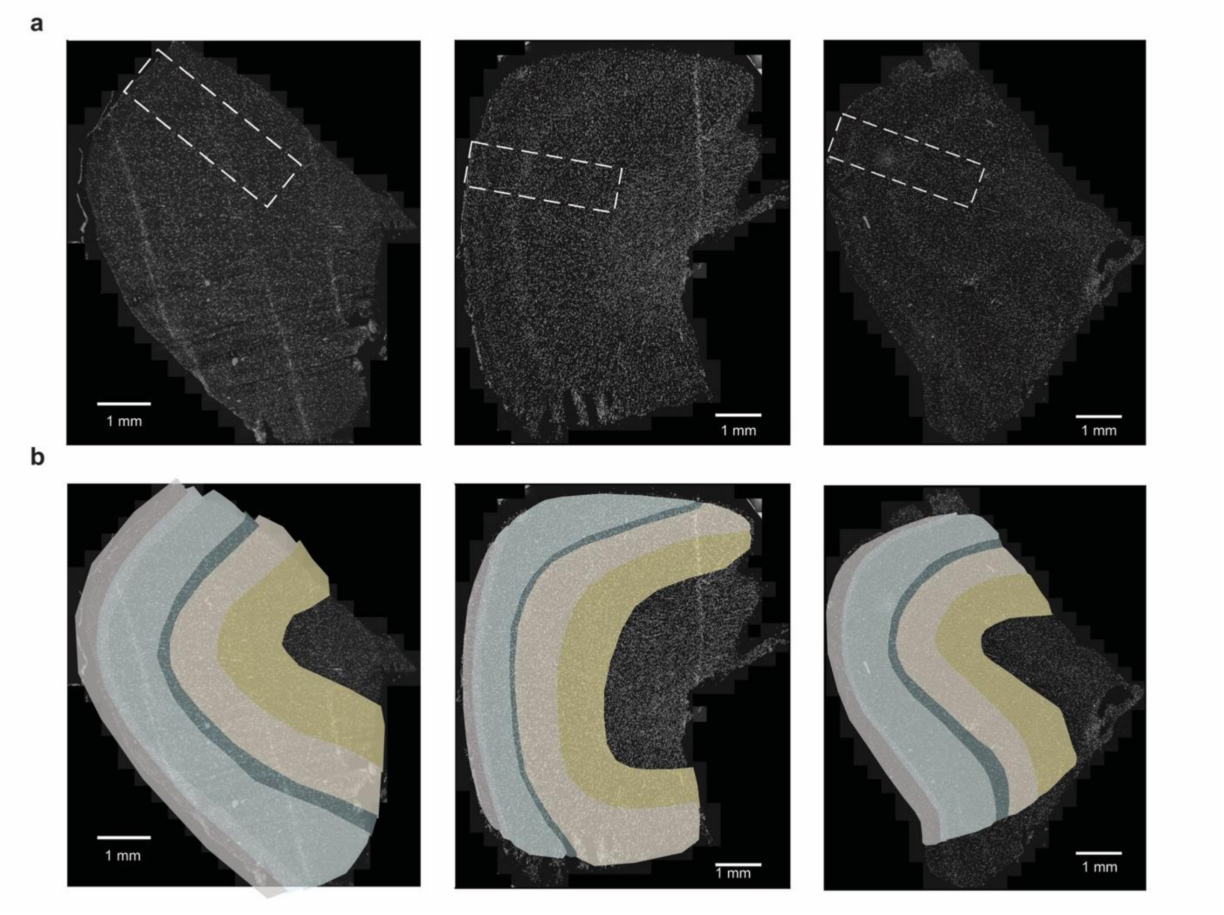

Supplementary Figure 5. Regions of interest and annotated neocortical layers in the human temporal lobe sections.

a, Region of interest on top of DAPI-stained sections for Sections A-C (left to right). **b,** Annotated neocortical layers on top of DAPI-stained sections for Sections A-C (left to right).

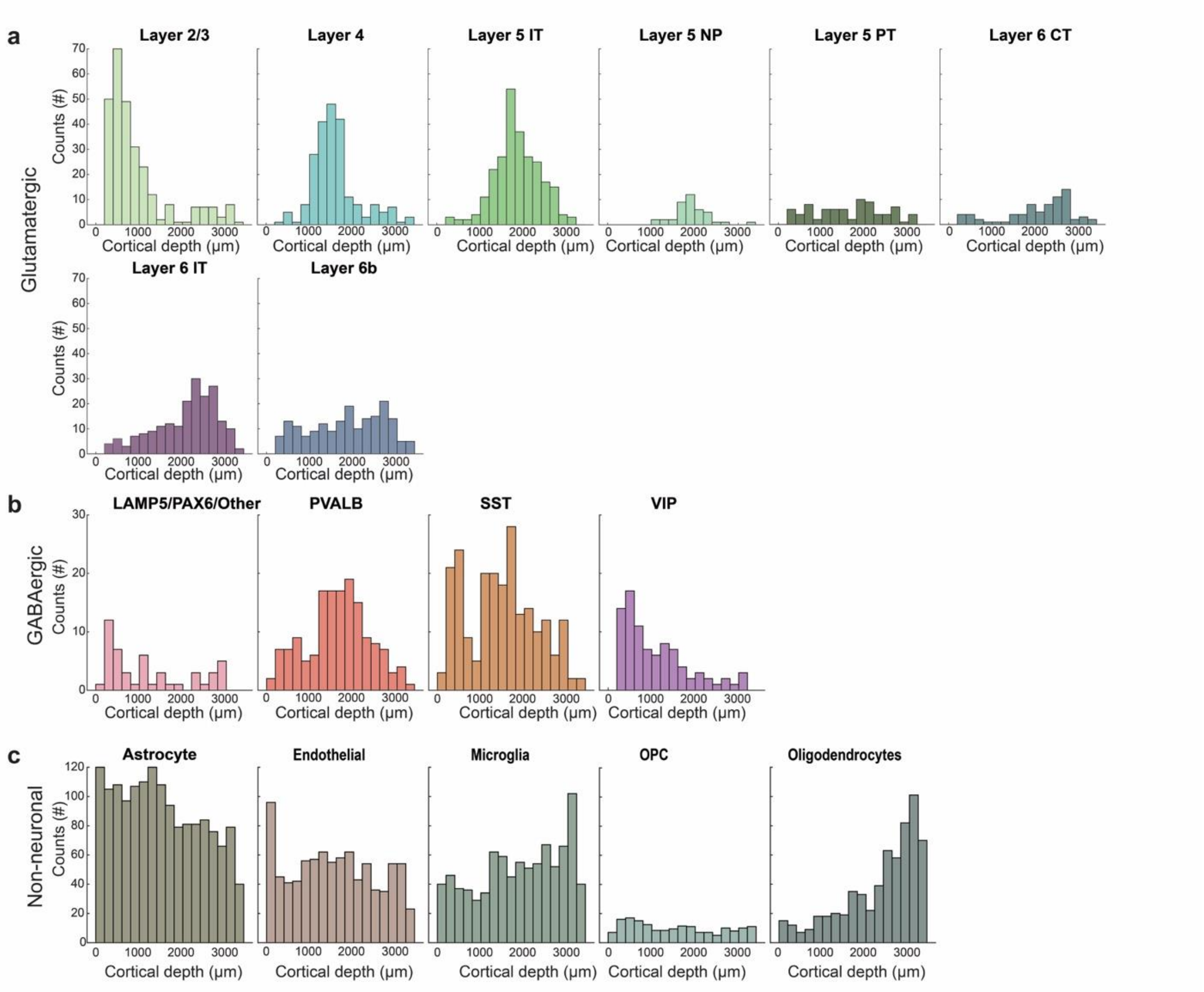

Supplementary Figure 6. Cortical profiles of glutamatergic, GABAergic and non-neuronal cells.

a, The cortical depth profile (1 bin = 200 μm) of the glutamatergic cell subclasses. **b,** Same as **a** for the GABAergic cell subclasses. **c,** Same as **a** for the non-neuronal cell subclasses.

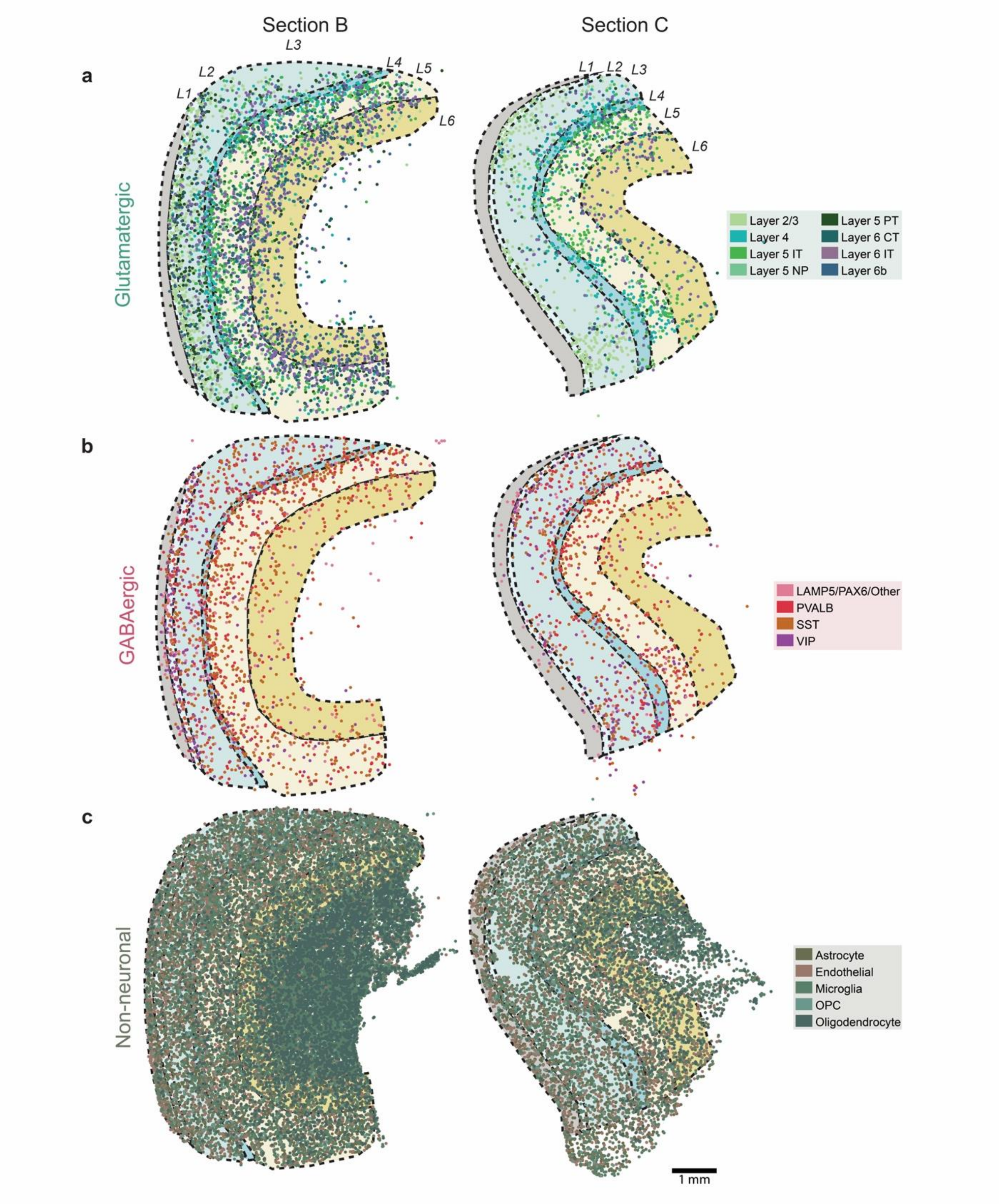

Supplementary Figure 7. Cell locations and annotated neocortical layers.

a, Location of glutamatergic cells colored by the most probable cell subclass (Section B (left) and C (right)). In the background, the annotated neocortical layers (L1-6) are plotted. **b,** Same as **a** for GABAergic cell subclasses. **c,** Same as **a** for non-neuronal cell subclasses.

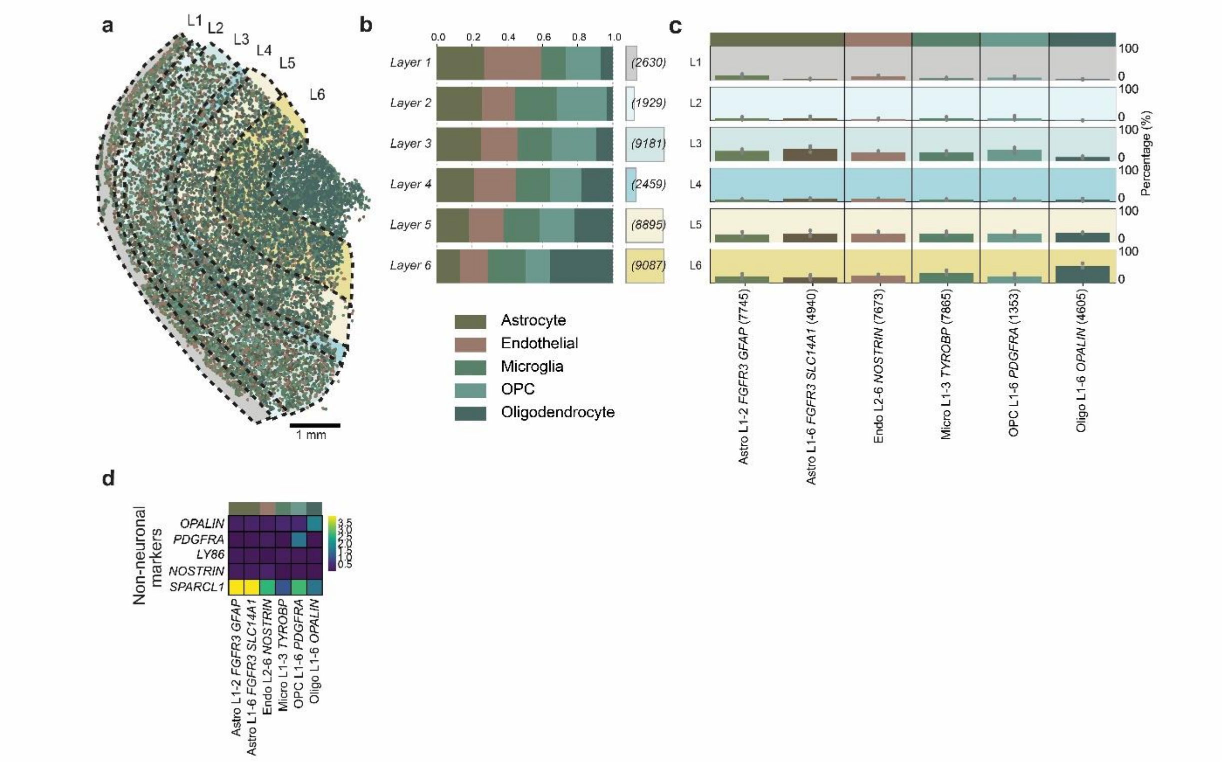

Supplementary Figure 8. Layer-specificity and molecular composition of non-neuronal cells.

a, Location of non-neuronal cells colored by the most probable cell subclass and annotated neocortical layers (L1-6). **b,** The within-layer relative distribution of non-neuronal cells and the number of cells counted for each layer in brackets. **c,** Across-layer distribution of non-neuronal cell types. The colored bars represent the relative proportion of each cell type in each layer (L1-6). Error bars represent standard error of the mean (n = 3 tissue sections). **d,** Mean log2-transformed expression of known non-neuronal marker genes.

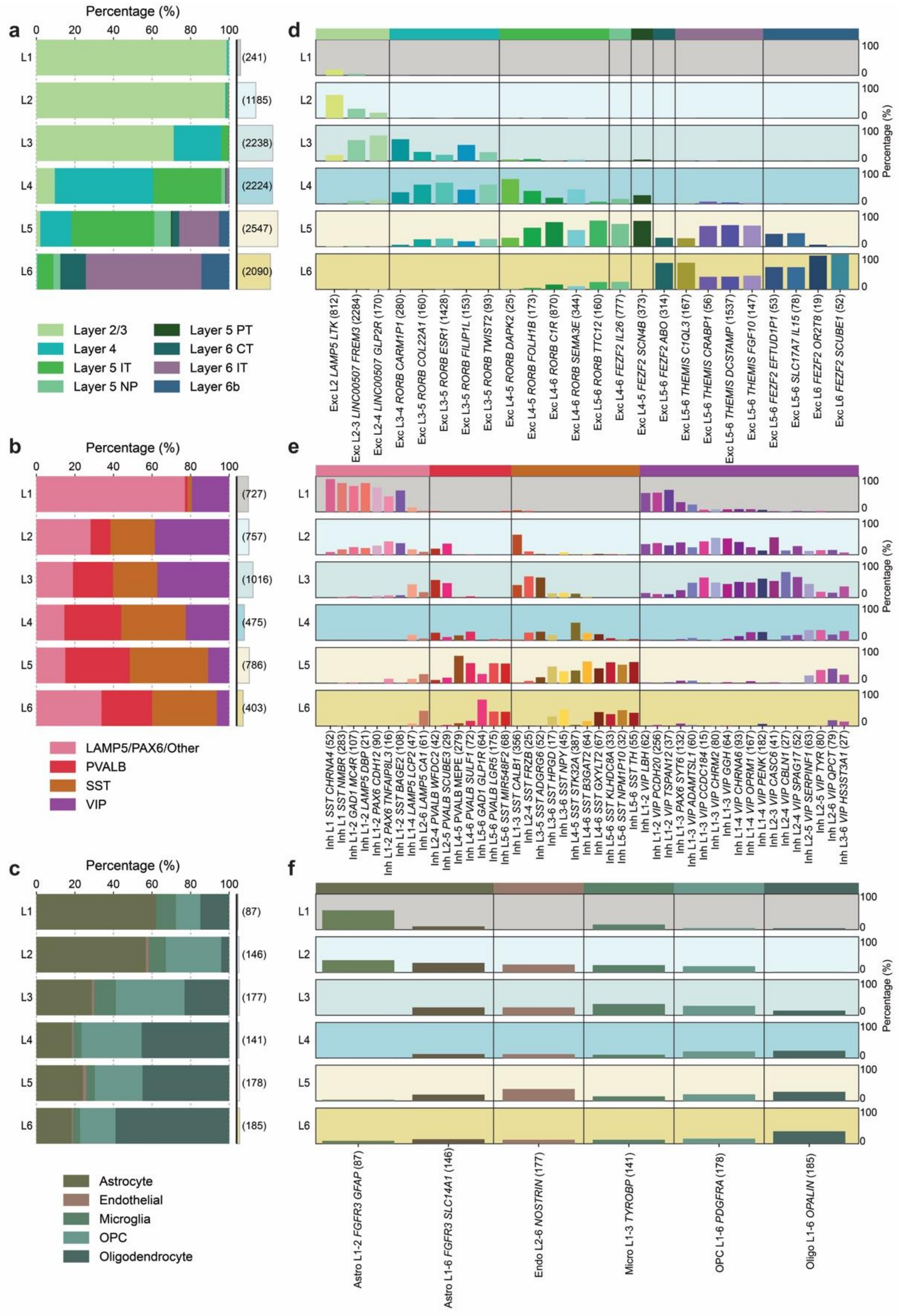
a
b
c
d
e
f
Percentage (%)
L1
L2
L3
L4
L5
L6
Layer 2/3
Layer 4
Layer 5 IT
Layer 5 NP
Layer 5 PT
Layer 6 CT
Layer 6 IT
Layer 6b
LAMP5/PAX6/Other
PVALB
SST
VIP
Astrocyte
Endothelial
Microglia
OPC
Oligodendrocyte
Exc L2 LAMP5 LTK (812)
Exc L2-3 LINC00507 FREM3 (2284)
Exc L2-4 LINC00507 GLP2R (170)
Exc L3-4 RORB CARM1P1 (280)
Exc L3-5 RORB COL22A1 (160)
Exc L3-5 RORB ESR1 (1428)
Exc L3-5 RORB FILIP1L (153)
Exc L3-5 RORB TWIST2 (93)
Exc L4-5 RORB DAPK2 (25)
Exc L4-5 RORB FOLH1B (173)
Exc L4-6 RORB C1R (870)
Exc L4-6 RORB SEMA3E (344)
Exc L5-6 RORB TTC12 (160)
Exc L4-6 FEZF2 IL26 (777)
Exc L4-5 FEZF2 SCN4B (373)
Exc L5-6 FEZF2 ABO (314)
Exc L5-6 THEMIS C1QL3 (167)
Exc L5-6 THEMIS CRABP1 (56)
Exc L5-6 THEMIS DCSTAMP (1537)
Exc L5-6 THEMIS FGF10 (147)
Exc L5-6 FEZF2 EFTUD1P1 (53)
Exc L5-6 SLC17A7 IL15 (78)
Exc L6 FEZF2 OR2T8 (19)
Exc L6 FEZF2 SCUBE1 (52)
Astro L1-2 FGFR3 GFAP (87)
Astro L1-6 FGFR3 SLC14A1 (146)
Endo L2-6 NOSTRIN (177)
Micro L1-3 TYROBP (141)
OPC L1-6 PDGFRA (178)
Oligo L1-6 OPALIN (185)

Supplementary Figure 9. Occurrences of glutamatergic, GABAergic and non-neuronal cells separated by layers in the human middle temporal gyrus from single-nucleus RNA-sequencing (snRNA-seq) data.

Single-nucleus RNA-sequencing (snRNA-seq) data from Hodge *et al* [1] **a,** The within-layer relative distribution of glutamatergic cells and the number of cells counted for each layer in brackets. **b,** Same as **a** for GABAergic cells. **c,** Same as **b** for non-neuronal cells. **d,** Across-layer distribution of glutamatergic cell types. The colored bars represent the relative proportion of each cell type in each layer (L1-6). **e,** Same as **d** for GABAergic cell types. **f,** Same as **d** for non-neuronal cell types.

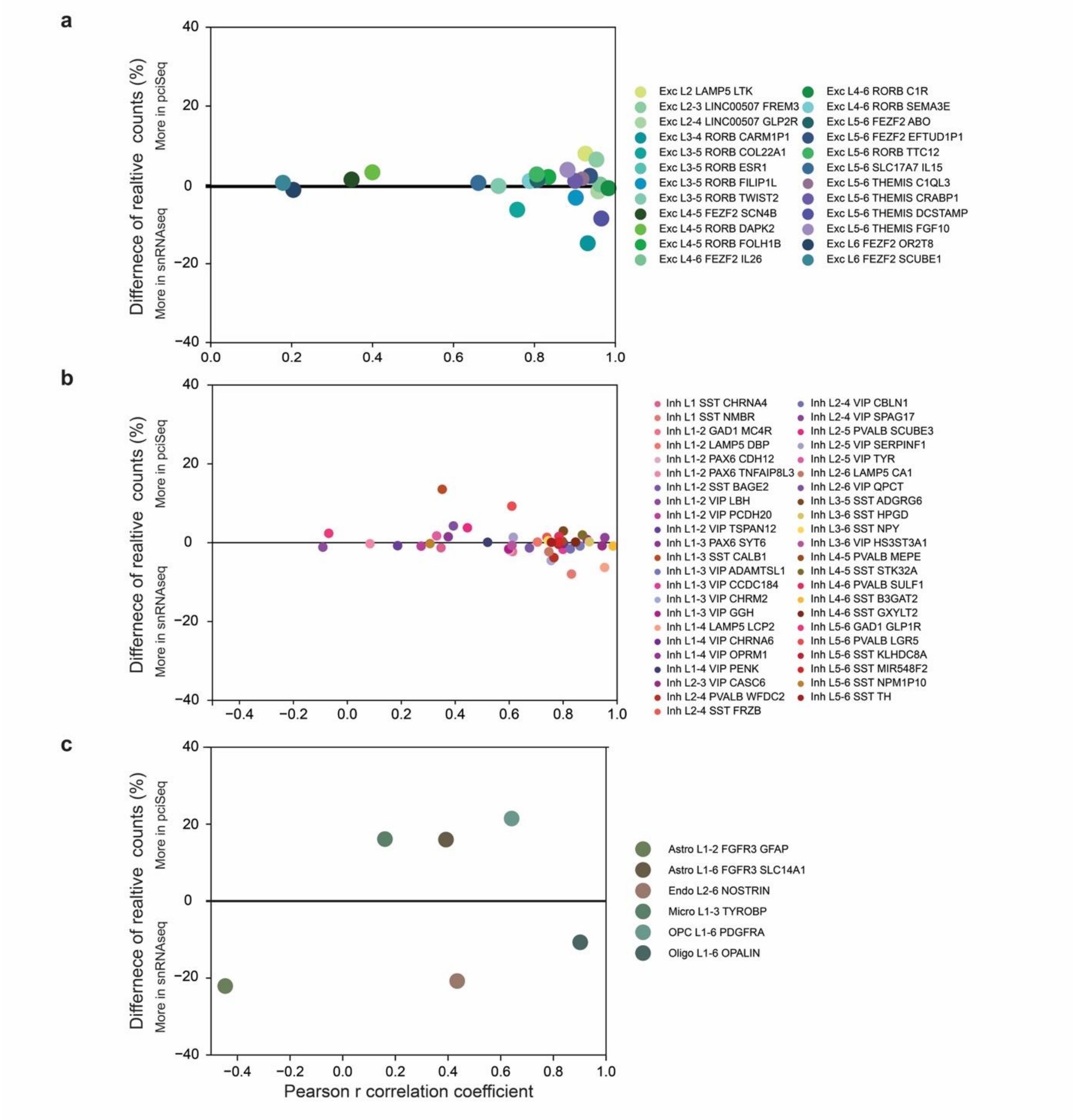

Supplementary Figure 10. Comparison of cell layer distribution and relative occurrences of cell types between pciSeq and snRNA-seq data.

a, Scatter plot of Pearson correlation coefficient on the X-axis and the difference in relative occurrence between pciSeq and snRNA-seq data on the Y-axis for the glutamatergic cell types. **b,** Same as **a** for GABAergic cell types. **c,** Same as **a** for non-neuronal cell types.

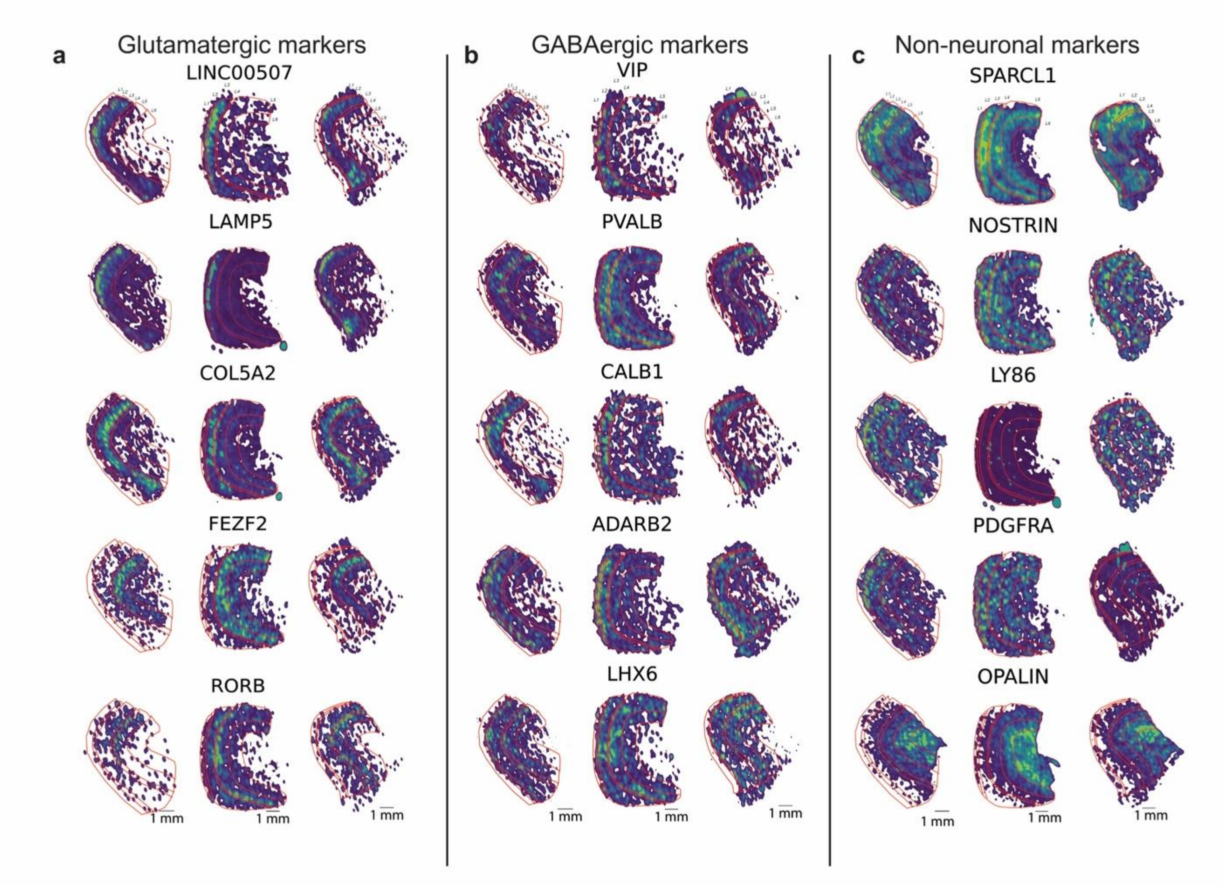

Supplementary Figure 11. Gene expression of known glutamatergic, GABAergic and non-neuronal marker genes.

a, Heatmap of the expression of known glutamatergic marker genes in the human temporal lobe sections. Layers are outlined in red to highlight the region specific expression. **b,** The same as **a** for GABAergic marker genes. **c** The same as **a** for non-neuronal marker genes.

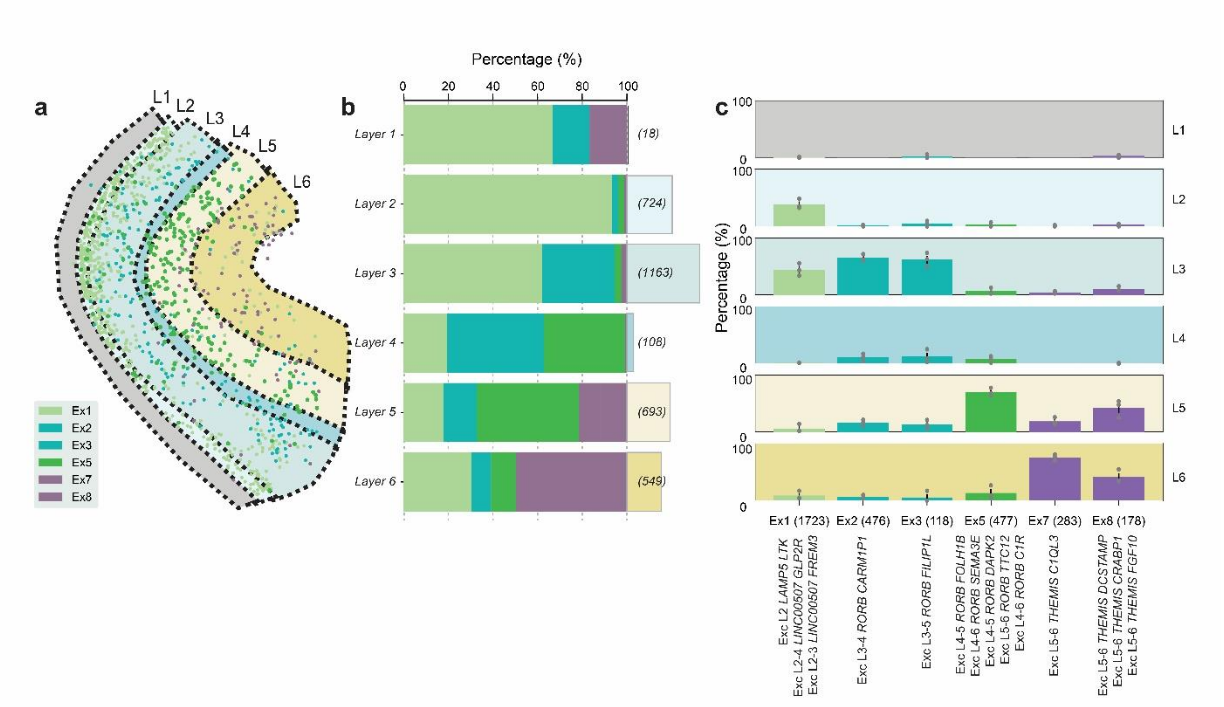

Supplementary Figure 12. Independent mapping of excitatory cell types with snRNA-seq data from Lake *et al.*[2].

a, Location of glutamatergic cells colored by the most probable cell subclass and annotated neocortical layers (L1-6). Three excitatory clusters (Ex2, Ex3, Ex7) reported by Lake *et al.,*[2] matched one-to-one and the remaining clusters mapped to multiple clusters in Hodge *et al.,*[1] (see **c**). **b,** The within-layer relative distribution of excitatory cells and the number of cells counted for each layer in brackets. **c,** Across-layer distribution of excitatory cell types. The colored bars represent the relative proportion of each cell type in each layer (L1-6) and the cell type matching between Lake *et al.,*[2] and Hodge *et al.,*[1] is shown below. Error bars represent standard error of the mean (n = 3 tissue sections).

Supplementary References

1. Hodge, R. D. *et al.* Conserved cell types with divergent features in human versus mouse cortex. *Nature* **573**, 61–68 (2019).
2. Lake, B. B. *et al.* Neuronal subtypes and diversity revealed by single-nucleus RNA sequencing of the human brain. *Science* **352**, 1586–1590 (2016).
