# Supplementary material for: Comprehensive in situ mapping of human cortical transcriptomic cell types
Source: Commun Biol. 2021 Aug 24;4:998. doi: 10.1038/s42003-021-02517-z (PMC8384853; doi:10.1038/s42003-021-02517-z)
Supplement: Supplementary file 2 — Description of Supplementary Files [file 42003_2021_2517_MOESM2_ESM.pdf]

Description of Additional Supplementary files

File name: Supplementary Data 1

Description: Cell metadata for the three tissue sections containing cell number, X coordinate, Y coordinate, annotated subtype, cell type probability, plot color, layer annotation, subclass, class, section.
