# Supplementary material for: Comprehensive in situ mapping of human cortical transcriptomic cell types
Source: Commun Biol. 2021 Aug 24;4:998. doi: 10.1038/s42003-021-02517-z (PMC8384853; doi:10.1038/s42003-021-02517-z)
Supplement: Supplementary file 4 — Reporting Summary [file 42003_2021_2517_MOESM4_ESM.pdf]

Corresponding author(s): Markus M. Hilscher, Mats Nilsson

Last updated by author(s): Jul 7, 2021

Reporting Summary

Nature Research wishes to improve the reproducibility of the work that we publish. This form provides structure for consistency and transparency in reporting. For further information on Nature Research policies, see our Editorial Policies and the Editorial Policy Checklist.

Statistics

For all statistical analyses, confirm that the following items are present in the figure legend, table legend, main text, or Methods section.

n/a	Confirmed	
☐	☒	The exact sample size (*n*) for each experimental group/condition, given as a discrete number and unit of measurement
☐	☒	A statement on whether measurements were taken from distinct samples or whether the same sample was measured repeatedly
☐	☒	The statistical test(s) used AND whether they are one- or two-sided<br>*Only common tests should be described solely by name; describe more complex techniques in the Methods section.*
☐	☒	A description of all covariates tested
☐	☒	A description of any assumptions or corrections, such as tests of normality and adjustment for multiple comparisons
☐	☒	A full description of the statistical parameters including central tendency (e.g. means) or other basic estimates (e.g. regression coefficient) AND variation (e.g. standard deviation) or associated estimates of uncertainty (e.g. confidence intervals)
☐	☒	For null hypothesis testing, the test statistic (e.g. *F*, *t*, *r*) with confidence intervals, effect sizes, degrees of freedom and *P* value noted<br>*Give P values as exact values whenever suitable.*
☒	☐	For Bayesian analysis, information on the choice of priors and Markov chain Monte Carlo settings
☒	☐	For hierarchical and complex designs, identification of the appropriate level for tests and full reporting of outcomes
☐	☒	Estimates of effect sizes (e.g. Cohen's *d*, Pearson's *r*), indicating how they were calculated

Our web collection on statistics for biologists contains articles on many of the points above.

Software and code

Policy information about availability of computer code

Data collection	Cell type definitions used in pciSeq were downloaded from the Allen Brain Atlas (https://portal.brain-map.org/atlases-and-data/rnaseq). All of the colors used in the figures follow the cell type color definition formulated by the Allen Institute.
Data analysis	The description of the image analysis can be found in Gyllborg et al.7 The same in-house software was used for the generation of the spatial gene expression data and is available at https://github.com/Moldia. The downstream processing of the data was done using a set of functions created in-house (https://github.com/Moldia/pciseq_processing_module). The set of tools included finding the most probable cell type call from the pciSeq output, plotting of these cell type calls, plotting the cell types individually, adding polygon labels to the cells and creating cell-by-gene matrices of the called cells.

For manuscripts utilizing custom algorithms or software that are central to the research but not yet described in published literature, software must be made available to editors and reviewers. We strongly encourage code deposition in a community repository (e.g. GitHub). See the Nature Research guidelines for submitting code & software for further information.

Data

Policy information about availability of data

All manuscripts must include a data availability statement. This statement should provide the following information, where applicable:

- Accession codes, unique identifiers, or web links for publicly available datasets
- A list of figures that have associated raw data
- A description of any restrictions on data availability

Raw data available upon request.

April 2020

Field-specific reporting

Please select the one below that is the best fit for your research. If you are not sure, read the appropriate sections before making your selection.

[x] Life sciences [ ] Behavioural & social sciences [ ] Ecological, evolutionary & environmental sciences

For a reference copy of the document with all sections, see nature.com/documents/nr-reporting-summary-flat.pdf

Life sciences study design

All studies must disclose on these points even when the disclosure is negative.

Sample size	*Describe how sample size was determined, detailing any statistical methods used to predetermine sample size OR if no sample-size calculation was performed, describe how sample sizes were chosen and provide a rationale for why these sample sizes are sufficient.*
Data exclusions	no data was excluded
Replication	Three tissue sections were independently mapped. Section A and C are from the middle temporal gyrus of a 38-year old male epilepsy donor. Section B is from the fronto-temporal parietal lobe of a 72-year old male tumor case. Sections are anonymized.
Randomization	n/A
Blinding	n/A

Reporting for specific materials, systems and methods

We require information from authors about some types of materials, experimental systems and methods used in many studies. Here, indicate whether each material, system or method listed is relevant to your study. If you are not sure if a list item applies to your research, read the appropriate section before selecting a response.

Materials & experimental systems

n/a	Involved in the study	
[x]	[ ]	Antibodies
[x]	[ ]	Eukaryotic cell lines
[x]	[ ]	Palaeontology and archaeology
[x]	[ ]	Animals and other organisms
[ ]	[x]	Human research participants
[x]	[ ]	Clinical data
[x]	[ ]	Dual use research of concern

Methods

n/a	Involved in the study	
[x]	[ ]	ChIP-seq
[x]	[ ]	Flow cytometry
[x]	[ ]	MRI-based neuroimaging

Human research participants

Policy information about studies involving human research participants

Population characteristics	Section A and C are from the middle temporal gyrus of a 38-year old epilepsy donor. Section B is from the fronto-temporal parietal lobe of a 72-year old male tumor case.
Recruitment	Human tissue was received from the Allen Institute for Brain Science under the SpaceTx consortium.
Ethics oversight	Tissue collection and processing was performed as previously described in Hodge et al. 2019, in accordance with the provisions of the United States Uniform Anatomical Gift Act of 2006 described in the California Health and Safety Code section 7150 (effective 1 January 2008) and other applicable state and federal laws and regulations.

Note that full information on the approval of the study protocol must also be provided in the manuscript.
